# Non-pharmacotherapeutic Management of Alcohol Use Disorder in the Alaska Native Population: A Narrative Review

**DOI:** 10.7759/cureus.39090

**Published:** 2023-05-16

**Authors:** Nikhil B Godbole, Udit Dave, Emma Lewis, Nupur Godbole, Gregory Sullivan, Autumn Schultz

**Affiliations:** 1 School of Medicine, Tulane University School of Medicine, New Orleans, USA; 2 Department of Psychiatry, University of South Florida Morsani College of Medicine, Tampa, USA

**Keywords:** substance use disorder (sud), native american, addiction disorder, alaska native, alcohol use disorder

## Abstract

Alcohol use disorder (AUD) is a leading preventable cause of death in the United States and has had a greater health impact on Alaska Natives than on any other racial group. To date, AUD in these communities has had wide-reaching negative impacts contributing to high rates of suicide, homicide, and accidents. A variety of genetic, experiential, social, and cultural factors have been associated with this trend. For decades, the Alaska Native subgroup has received inadequate treatment. The purpose of this review is to evaluate current trends in effective interventions and to help answer the question: What may comprise a successful non-pharmacotherapeutic interventional strategy to treat and prevent AUD in Alaska Natives? A database literature search was performed in September 2022 using the PubMed library. Search terms included (alcohol use disorder) AND ((Alaska OR Alaskan) Native). Inclusion criteria included full-text articles, a focus on specific non-pharmacotherapeutic treatment strategies, and a publication date after 2005. Studies that did not evaluate non-pharmacotherapeutic interventions, evaluated a population other than Alaska Natives, evaluated a disorder other than AUD, were written in a language other than English, or were editorials or opinion pieces were excluded. The selected studies were assessed for bias utilizing the Newcastle-Ottawa Scale (NOS). Twelve studies were included in this review. This review found that early social network intervention, incentive-driven programs, culturally-driven programs, and motivational interviewing are promising non-pharmacotherapeutic interventions in the treatment of AUD in Alaska Native communities. Evidence suggests that a shift in focus to the accentuation of protective factors and the mitigation of isolation as a risk factor, rather than on the reduction of more intractable risk factors, may be associated with improved outcomes in treating AUD. The literature also suggests that successful prevention strategies should be driven by indigenous knowledge and grounded in community and culture. This study has its limitations. These include a lack of direct comparisons between studies, a lack of pooled statistical analysis or synthesis, and a lack of quantitative analysis. Instead, the majority of data is gathered from more bias-prone cross-sectional studies and, thus, should be used to provide insight into potential risk factors and non-pharmacologic therapies effective in this population rather than as strong evidence in favor of one therapeutic regimen over another. For this, there is a need for more clinical trials evaluating treatments for AUD in this population. This review received support from the University of South Florida Department of Psychiatry. There were no sources of funding for this work from any institution. There are no competing financial or non-financial interests that may be interested in this work. This review is not registered. This review does not have a prepared protocol.

## Introduction and background

Alaska Natives comprise a diverse community. Today, Alaska Natives make up 16% of the state’s population [1]. Despite this, Alaska Natives account for a majority of alcohol-related hospital admissions in the state when compared to all other races [2]. These tribes primarily belong to five distinct native groups. Southeast Alaska, with its mild coastal climate, is home to the Tlingit, Haida, and Tshimshian groups, while the state’s much harsher interior is home to the Athabascan groups. The northern and northwestern coastal region was primarily settled by the Inupiaqs, while the Yupiks settled within southwest Alaska. The smallest group of Alaska natives, the Aleuts, is native to the Aleutian islands straddling the westernmost fringes of the state [1].

It is well known that chronic alcohol use has myriad systemic manifestations, most commonly affecting the liver but also every organ system in the body [3]. Notably, the range and severity of these adverse effects have a sizable genetic component [4]. As such, genetic predisposition is a risk factor in the development of alcohol use disorder (AUD), and natural variants in genotypes encode a range of activities of the alcohol dehydrogenase and aldehyde dehydrogenase enzymes, which are responsible for alcohol metabolism [4]. Studies of allelic frequency among five distinct Native Alaskan groups - Yupik, Inupiaq, Athabascan, Tlingit, and Aleut - revealed that there are no generalizable differences between Alaska Natives with alcohol dependence and those in the general population [5]. Non-genetic risk factors for AUD include the amount of alcohol consumed, age when drinking first began, family history of alcoholism, level of education, gender, and prenatal exposure [6].

AUD remains undertreated, contributing to significant psychosocial and public health consequences [7]. Using the DSM-5 diagnostic criteria and according to the American Psychiatric Association, current treatment guidelines state initial treatment should include goal setting for alcohol reduction and harm management in addition to exploring pharmacotherapy options [7]. For example, naltrexone, disulfiram, or acamprosate may be offered as a first-line treatment to reduce cravings [7]. Non-responders to this therapy can be prescribed second-line agents such as topiramate or gabapentin. The non-pharmacotherapeutic standard of care is cognitive-behavioral therapy, twelve-step therapy, and motivational interviewing [7]. Within the Alaska Native community, this issue is of particular urgency.

Over the course of history, the classification and diagnosis of AUD have changed, and it begs the question, did this impact reporting, and how [8]? The DSM-III and its revision loosely referred to addiction but primarily focused on dependence. The DSM-IV was able to expand on the addiction process outside of dependence. Currently, the DSM-5 notably contains the addition of cravings, removal of the criteria of legal problems, and the consolidation of the diagnoses from abuse and dependence [9].

## Review

Methods

Article Search and Selection

A literature search of the PubMed database was conducted in September 2022 following PRISMA guidelines [10] using the search term (alcohol use disorder) AND ((Alaska OR Alaskan) Native) to evaluate the non-pharmacologic management of AUD in Alaska Natives. Inclusion criteria included full-text articles, a focus on specific non-pharmacotherapeutic treatment strategies, and a publication date after 2005. Studies that did not evaluate non-pharmacotherapeutic interventions, evaluated a population other than Alaska Natives, evaluated a disorder other than AUD, were written in a language other than English, or were editorials or opinion pieces were excluded. Articles were classified independently by two reviewers (NG and UD), and any discrepancies were resolved by a third reviewer (EL).

Qualitative Analysis

The Newcastle-Ottawa Scale (NOS) [11] was used for risk of bias assessment. The NOS criteria allowed for a maximum of four stars in the selection, two stars in comparability, and three stars in the outcome: the total range was 0-9 for randomized control trials and cohort studies. The total range was 0-8 for cross-sectional studies. Cohort studies, randomized controlled trials, and cross-sectional studies were analyzed with respective NOS guidelines.

**Table 1:** NOS qualitative analysis of study risk NOS: Newcastle-Ottawa Scale, RCT: randomized controlled trial

Table 1: NOS qualitative analysis of study risk										
		Selection				Comparability	Outcome			
Cohort studies										
Author and year	Study type	Representativeness of the exposed ​​cohort	Selection of the non-exposed cohort	Ascertainment of ​exposure	Demonstration that outcome of interest was not present at the start of the study	Comparability of cohorts on the basis of the design or analysis	Assessment of ​​outcome	Was follow-up long enough for outcomes to occur	Adequacy of follow-up of cohorts	Total
Beckstead, 2015	​​Cohort	1	1	1	1	0	1	1	1	7
Randomized controlled trial										
		Selection				Comparability	Outcome			
Author and year	Study type	Is the case definition adequate	Representativeness of the cases	Selection of controls	Definition of controls	Comparability of cases and controls on the basis of the design or analysis	Ascertainment of exposure	The same method of ascertainment for cases and controls	Non-response rate	Total
McDonell, 2021	RCT	1	1	1	1	0	1	1	1	7

						Comparability	Outcome		
Author and Year	Study type	Representativeness of the exposed ​​cohort	Sample size	Non-respondents	Ascertainment of the exposure (risk factor)	Comparability of cohorts on the basis of the design or analysis	Assessment of ​​outcome	Statistical test	Total
Lillie, 2021	Cross-sectional	1	1	1	1	0	1	1	6
Philip, 2016	Cross-sectional	1	1	1	1	0	1	1	6
Seale, 2006	Cross-sectional	1	1	1	1	0	1	1	6
Hirchak, 2018	Cross-sectional	1	1	1	1	0	1	1	6
Dickerson, 2016	Cross-sectional	1	1	1	1	0	1	1	6
Allen, 2014	Cross-sectional	1	1	1	1	0	1	1	6
Skewes, 2016	Cross-sectional	1	1	1	1	0	1	1	6
Mohney, 2022*	Book chapter	-	-	-	-	-	-	-	-
Emerson, 2019*	Review	-	-	-	-	-	-	-	-
Rasmus, 2019*	Review	-	-	-	-	-	-	-	-

Results

**Figure 1:**
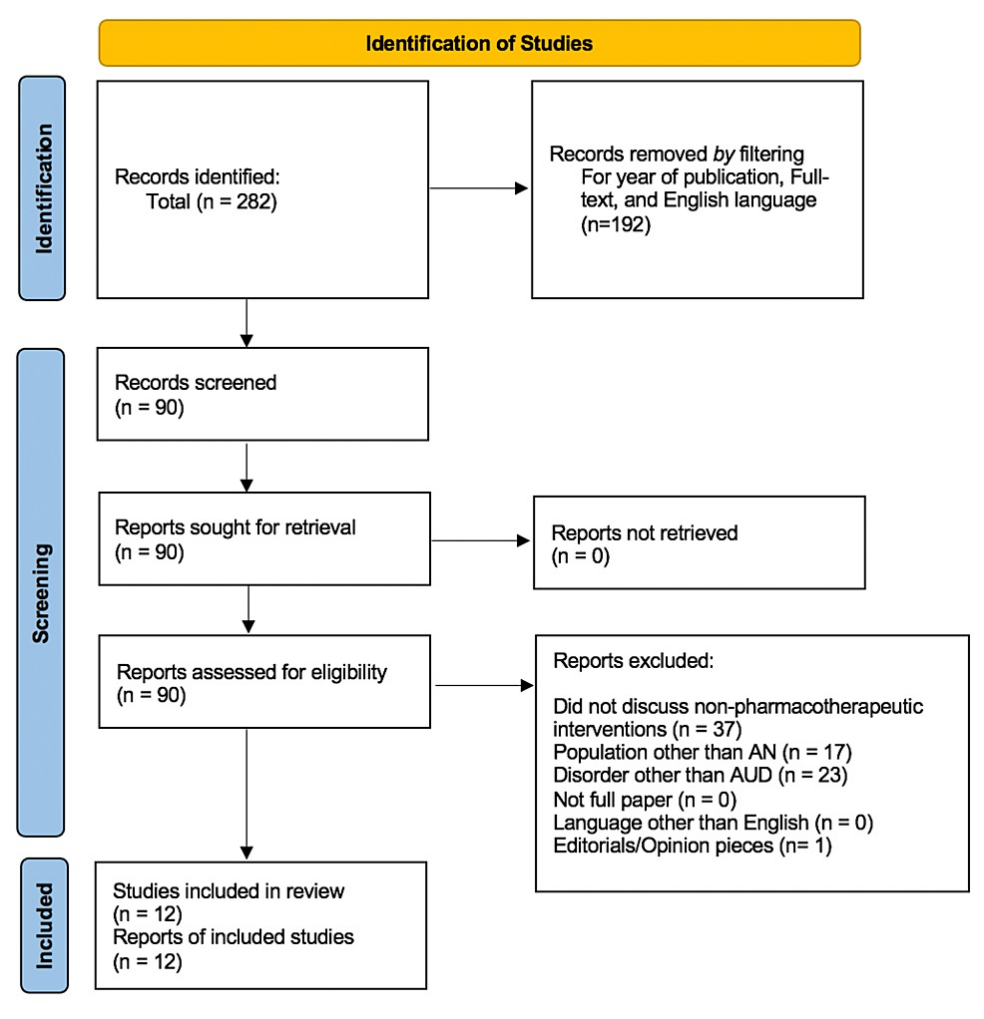
Article selection flow diagram

An initial keyword search identified 282 articles for inclusion. A filter was applied to select for publication year, English language, and full text, leaving 192 articles for screening. Of these articles, 78 were excluded according to exclusion criteria, leaving 12 articles for further assessment. These 12 included articles included one cohort study, one randomized controlled trial, seven cross-sectional studies, two reviews, and one book chapter (Figure 1). Data collection was conducted by a single reviewer (NG). In data collection, particular focus was placed on historical elements of AUD in Alaska Natives, psychosocial risk factors for AUD in Alaska Natives, and interventions for AUD in Alaska Natives.

Qualitative assessment

The studies were assessed with appropriate guidelines to characterize their quality based on several criteria (Table 1). NOS scores of 7-9 were deemed sufficient for randomized controlled trials and cohort studies as it fell in the region of “high quality” study according to the NOS guidelines [11]. NOS scores 6-8 were deemed sufficient for cross-sectional studies. All 12 articles were within this range.

Risk factors for alcohol use in Alaska Natives

Alaska Natives experience a steadily increasing burden of alcohol use that far exceeds that of other American populations such as non-native Alaskans and Native Americans on the mainland US [12]. Research seeking to identify etiologies of alcohol use specific to Alaska Natives identified several criteria including genetics, traumatic exposure, home environment, peer interactions, and sociocultural factors [12].

Deficiencies in socialization coupled with negative-effect regulation have been implicated in the pathogenesis of AUD [8]. The introduction of alcohol into Alaska Native communities is relatively new, as these populations had no exposure to alcohol prior to contact with European colonizers [2]. Federal assimilation programs also created “lost generations” by forcibly removing children from their homes and placing them in boarding schools in which they were punished for speaking their native languages [2]. The denial of cultural customs has been attributed to impeding the successful development of native youth [2]. As such, social isolation coupled with geographic isolation may play a role in the pathophysiology of AUD within the Alaska Native subgroup.

Interventions to address alcohol use in Alaska Native communities

Today, excessive alcohol consumption is a leading preventable cause of death in the United States, disparately harming indigenous groups more severely than any other racial group [13]. Historically, many restrictive interventions have been implemented in an effort to reduce the alcohol use discrepancy faced by Alaska Natives. Alcoholic beverages have been banned by popular vote in many native villages [2]. Although proven to provide safety benefits, namely, with regard to alcohol-related violence, these local option laws remain controversial in their efficacy at preventing AUD due to the limited benefits they offer [2].

The literature currently identifies social network interventions, incentive-driven programs, culturally driven programs, and motivational interviewing as front-line non-pharmacologic interventions to simultaneously address isolation (risk factor) and accentuate community and familial engagement (protective factor).

A 2015 study on Yupik youth identified social network intervention as a promising systemic solution for AUD [14]. This study posits that increasing the density and closeness of family and elder ties could function as a protective factor against AUD [14]. A qualitative behavioral study on the Inupiaq revealed that in many communities, there exists a dissociation between a desire to live according to traditional subsistence practices preached by elders and a pressure to attend school and enter the wage economy [15]. This dissonance leads to many feelings of decreased self-worth and lack of direction [15]. Such positive intervention programs would ideally include services for high-risk youth and incorporate family treatment with the goal of building multigenerational legacies focused on promoting subsistence traditions [15]. Essentially, returning to the traditional Alaska Native community-oriented approach toward healing personal and family problems may bring about immense benefits in treating and preventing AUD [15]. Additionally, chronic alcoholic liver disease has been associated with initiating alcohol use at an early age with subsequent increases in consumption with age [16]. As a result, this technique may allow for early intervention and prevention to encourage abstinence until age appropriate and enhance protective factors.

Incentive-driven approaches centered around operant conditioning principles have also demonstrated success. Contingency management is a feasible low-cost approach to AUD treatment that utilizes positive reinforcers to promote and support abstinence. These abstinence programs endeavored to foster family relationships and integrate indigenous languages and practices, many of which have been lost due to forced assimilation [17]. This method is advantageous in the ability to customize rewards to uphold practical and cultural standards. A 2018 mixed-methods randomized controlled trial found focus groups prior to contingency management to be most effective when prizes are practical, implemented in a community context, and able to foster familial engagement [16]. This finding is corroborated by a recent randomized controlled trial of 158 Alaska Native adults diagnosed with AUD. In this study, cultural incentive-driven abstinence programs were identified as promising interventions [17]. Cultural incentives included gift cards for local businesses, fishing equipment, and local artwork [17]. 

Evidence for the prospective success of culturally driven therapy is the current use of tribal traditional healing. This therapy is advantageous within the Alaska Native subgroup due to its inherent emphasis on spirituality [18]. As a result, it is sought by many Alaska Natives and used regularly. One solution may be to merge biomedical treatment with traditional tribal healing methodology in an effort to provide holistic care that incorporates physical wellness with spiritual and cultural wellness as well. The concept of positive intervention with both biological and tribal healing was explored by a pilot study that combined dialectical behavioral therapy with traditional cultural treatment [19]. This study revealed a high rate of improvement among adolescents [19], which has been the key target demographic for early intervention strategies.

Motivational interviewing is another evidence-based therapy deemed consistent with Alaska Native values [20]. Studies suggest that wider dissemination of motivational interviewing within tribal treatment regimens may provide therapeutic benefits [20]. This is a patient-focused technique consisting of both a relational and technical aspect. The relational component consists of the interviewer establishing patient trust and rapport. The technical component then bolsters the individual’s motivation for change and reinforces their decision [18]. Young urban patients diagnosed with AUD who engaged with this study credited both internal and external motivation in bringing about positive change in their lives [9].

Discussion

This review strongly corroborates the efficacy of an indigenous knowledge-driven and strengths-based intervention framework grounded in community and culture in addition to risk factor reduction for AUD. The implementation of indigenously informed and locally sustainable strategies is critical in the promotion of health and well-being, especially in the field of AUD treatment. Recent studies have termed this paradigm the “Qasgiq Model” of community intervention [21]. Historically a round extended kinship shelter, Qasgiq has come to represent the interdependent circle of family and community [21]. Under this model, early family and community intervention serves to provide skills, cultural strength, and values that act as protective factors [21]. Furthermore, increased participation in cultural activities and traditions may provide increased purpose and meaning to alcohol-dependent adults.

However, the complexities of regional and demographic variation may require a broader range of therapeutic approaches or only specific interventions to be implemented. Additional studies must explore cultural mechanisms and variations in alcohol use within subgroups, such as the Athabascan, Tlingit, and Aleut populations. Furthermore, it is important to understand the granularity of behavioral patterns in these different groups to most effectively provide AUD care on both a population-specific and individualized basis.

Of note, there is a paucity of research examining drinking behavior in segments of the Alaska Native population, especially elders. This may partially be explained by a general distrust of behavioral health research following the Barrow Alcohol Study, notable for its ethics violations in cross-cultural research [22]. This study led to the propagation and widespread acceptance of the “intoxicated Indian” stereotype. Current treatment efforts should therefore aim to actively dismantle this myth. These studies underscore the importance of a collaborative and participatory framework for research and healthcare institutions. This requires fostering trusting relationships with community partners and appropriately contextualizing alcohol-related research results with respect to demographic and geographic factors [22].

Ultimately, research questions and treatment approaches should reflect local cultures, values, and experiences. In keeping with this approach, community partners have advocated for shifting to a strengths-based approach. This entails evaluating successful outcomes to accentuate protective factors in addition to addressing amenable risk factors [23]. Implementing such interventions in Alaska Natives may be especially worthwhile because despite having the highest levels of alcohol use, this population also seeks treatment for AUD at the highest rate of any other ethnicity [24]. As a result, there is great potential for improvement of AUD care in Alaska Native communities if they are provided with access to culturally competent specialized psychiatric care.

Limitations of this literature review include factors influencing epidemiological rates over an individual’s life span. This includes but is not limited to recovery with and without treatment, maturing out of AUD in those surviving to older age, incarceration for AUD-related crimes, death due to AUD-related causes, and moving out of Alaska to another state. All of the aforementioned factors limit the ability to accurately assay prevalence among age groups, and thus such comparisons have been excluded from this analysis. Another shortcoming is the presence of cultural heterogeneity within the Alaska Native subgroup. The standard reporting of Alaska Natives as an entire population is subject to misinterpretation and overgeneralization. Differences in language, customs, and culture lead to a large variance in alcohol use among tribes and geographical locations [18].

As data in this field is limited, we can draw attention to how the change in DSM criteria for AUD has correlated with research on this population over the years. It appears that rates of diagnosis of AUD among natives had been increasing even prior to the change of the DSM language in 1994, but since then, more data has been collected on help-seeking behaviors and changing the approach in lieu of a culturally contextualized one [25]. More recently, data shows that Alaska Native populations with AUD, being male, and aged 35-64 were statistically significant correlates of seeking treatment or help for AUD [24]. Based on older studies, there is scant data indicating the prevalence of seeking treatment for AUD among Alaska Natives, but much of the literature focuses on violent death and adverse outcomes rather than also focusing on rates of help-seeking in this subpopulation.

From the DSM-IV to the DSM-5, the addition of cravings to the diagnostic criteria for AUD may be thought of as a perceived beneficial change as it incorporates physiological aspects of addiction, outside of tolerance and withdrawal, into the definition. This change allows for open discussion techniques to decrease use for physiological benefit rather than focusing on harm reduction counseling [8]. Removing the criteria of legal problems specifically may also introduce a wider selection of individuals with cognitive, functional, and socioeconomic protective factors [8]. The consolidation of the diagnoses from abuse and dependence to AUD also appears to lessen the stigma associated with the volitional activity of consuming alcohol and instead allows us to place the patient on a continuum of pathologic severity and give significance to the non-modifiable risk factors that do exist [8]. Additionally, the change from polysubstance dependence to each individual named disorder allows each disorder to be treated as its own entity and, therefore, more appropriately treated according to specific evidence.

Important methodological limitations of this review include the reliance on primarily cross-sectional studies for data collection. The majority of works on this topic contain analysis of qualitative data with variation in outcome measures. There is also variation within evaluated exposures, as well as heterogeneity among chosen group demographics resulting in an inability to conduct higher-order statistical syntheses of results. This renders comparison among studies in the form of a systematic review difficult. This also results in a lack of quantitative effect measures and summary statistics regarding treatment efficacy. Although all included studies were qualitatively assessed as “high quality,” they all notably lacked comparability (Figure 1). As such, this increases the overall risk of bias. As a result, this body of evidence has considerably less certainty when compared to forms of more objective quantitative analysis. Furthermore, this body of work cannot be used to advocate for the efficacy of one treatment regimen over another. Instead, this work should be used as a framework to highlight important risk factors and current trends in effective non-pharmacologic treatment measures.

## Conclusions

Treatment of AUD in the Alaska Native subgroup remains an urgent issue. This study finds that multifaceted approaches encompassing early social network intervention, incentive-driven programs, culturally driven programs, and motivational interviewing may be efficacious as front-line non-pharmacotherapeutic interventions in the treatment of AUD in Alaska Native communities. The literature also finds that treatment efforts that are elder-led and indigenous-knowledge-driven may be associated with improved outcomes. Furthermore, a focus should be placed on mitigating isolation as a risk factor while accentuating protective factors. It is important to note that many risk factors associated with AUD are a product of systemic manipulation that will likely require more than social intervention programs to rectify. While a broad range of therapies is likely required, we advocate for the exploration of positive intervention programs with pharmacologic and nonpharmacologic treatment strategies that have shown to be efficacious in treating Alaska Natives struggling with AUD.

## Figures and Tables

**Figure 2:**
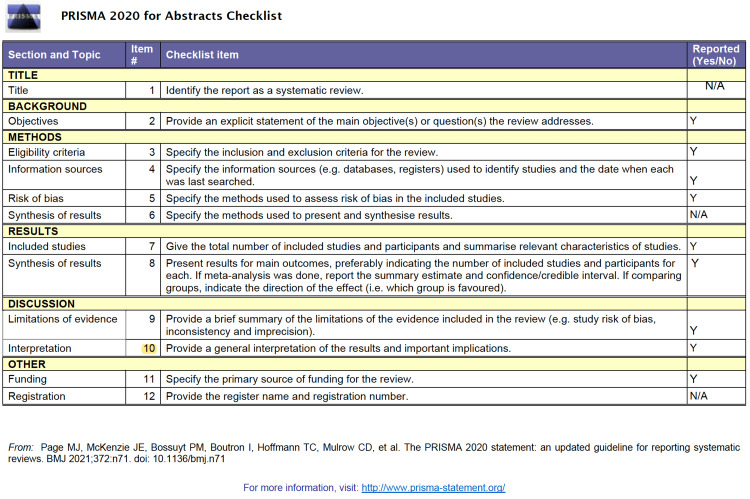
PRISMA 2020 checklist for abstracts

**Figure 3:**
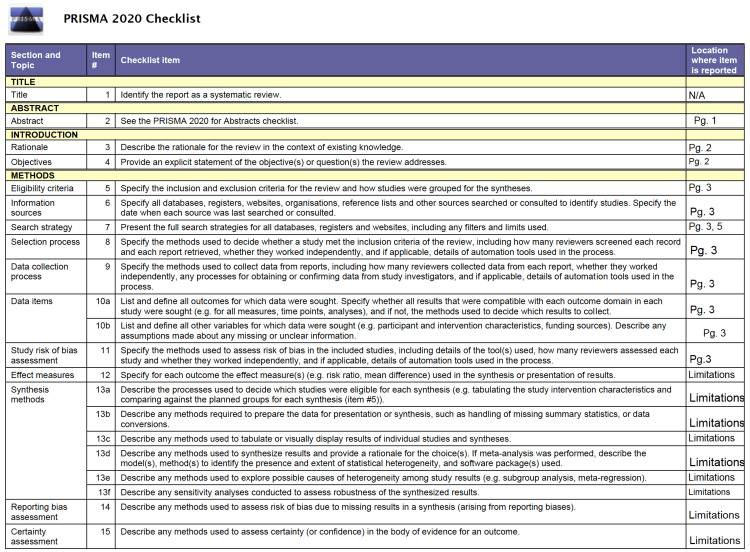
PRISMA 2020 checklist (title-methods)

**Figure 4:**
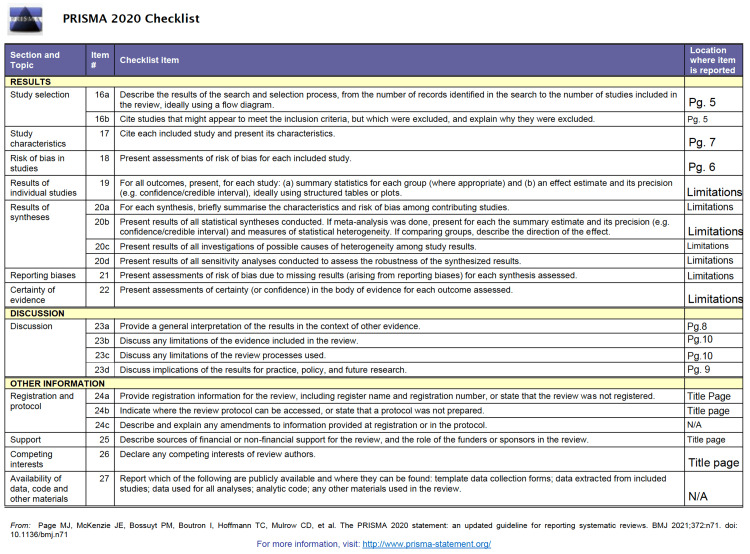
PRISMA 2020 checklist (results-other)
